# The spectrum of neurological presentation in individuals affected by *TBL1XR1* gene defects

**DOI:** 10.1186/s13023-024-03083-3

**Published:** 2024-02-20

**Authors:** Amanda Nagy, Francine Molay, Sarah Hargadon, Claudia Brito Pires, Natalie Grant, Lizbeth De La Rosa Abreu, Jin Yun Chen, Precilla D’Souza, Ellen Macnamara, Cynthia Tifft, Catherine Becker, Claudio Melo De Gusmao, Vikram Khurana, Ann M. Neumeyer, Florian S. Eichler

**Affiliations:** 1 Department of Neurology, Massachusetts General Hospital, 55 Fruit St, Wang Ambulatory Care Center 708, Boston, MA 02114 USA; 2 Division of Clinical Research, Massachusetts General Hospital, 55 Fruit St, Boston, MA 02114 USA; 3 Fly Little Bird Foundation, PO Box 698, Excelsior, MN 55331 USA; 4 National Human Genome Research Institute, National Institutes of Health, 10 Center Drive, Bethesda, MD 20892 USA; 5 Division of Movement Disorders, Department of Neurology, Brigham and Women’s Hospital and Harvard Medical School, Hale Building for Transformative Medicine Room 10016L, 60 Fenwood Road, Boston, 02115 USA; 6 Lurie Center for Autism, Massachusetts General Hospital, 1 Maguire Road, Lexington, MA 02124 USA

**Keywords:** TBL1XR1, Pierpont syndrome, Genetic diseases, Autism, Intellectual disability, Behavior, Epilepsy

## Abstract

**Background:**

*TBL1XR1* encodes a F-box-like/WD40 repeat-containing protein that plays a role in transcription mediated by nuclear receptors and is a known genetic cause of neurodevelopmental disease of childhood (OMIM# 608628). Yet the developmental trajectory and progression of neurologic symptoms over time remains poorly understood.

**Methods:**

We developed and distributed a survey to two closed Facebook groups devoted to families of patients with *TBL1XR1*-related disorder. The survey consisted of 14 subsections focused upon the developmental trajectories of cognitive, behavioral, motor, and other neurological abnormalities. Data were collected and managed using REDCap electronic data capture tools.

**Results:**

Caregivers of 41 patients with a *TBL1XR1*-related disorder completed the cross-sectional survey. All reported variants affecting a single amino acid, including missense mutations and in-frame deletions, were found in the WD40 repeat regions of Tbl1xr1. These are domains considered important for protein–protein interactions that may plausibly underlie disease pathology. The majority of patients were diagnosed with a neurologic condition before they received their genetic diagnosis. Language appeared most significantly affected with only a minority of the cohort achieving more advanced milestones in this domain.

**Conclusion:**

*TBL1XR1*-related disorder encompasses a spectrum of clinical presentations, marked by early developmental delay ranging in severity, with a subset of patients experiencing developmental regression in later childhood.

**Supplementary Information:**

The online version contains supplementary material available at 10.1186/s13023-024-03083-3.

## Background

Neurodevelopmental disorders such as autism spectrum disorder (ASD), intellectual disability (ID), and developmental delay, are common neurological conditions affecting the typical developmental trajectory [1]. With the rise of readily available genetic testing and the increasing awareness of treatable causes for ID [2], the recognition of genetic etiologies among these common conditions is increasing, including the identification of monogenic etiologies [3, 4]. Further, next generation sequencing techniques have allowed for discovery of new disease-causing genes, necessitating re-analysis of exome data over time. Despite these advances, many such genes have not been fully characterized in the literature and the spectrum of symptoms that should trigger genetic testing is often incomplete. There is thus need to further characterize and systematically chart symptom progression of these conditions that are typically rare. Moreover, disease-specific genetic panels, frequently used for directed testing, vary in the genes included and may include genes with limited evidence of association with disease [5]. Further, many genetic disorders include clinical features found on multiple panels, and it is not always clear to which panels these genes should be added.

One such gene is transducin (beta)-like X-linked receptor 1 (*TBL1XR1*). Located on the long arm of chromosome 3, *TBL1XR1* encodes a F-box-like/WD40 repeat-containing protein Tbl1xr1 that acts as a transcriptional regulator as part of the nuclear receptor corepressor (NCoR)*/*silencing mediator of retinoic acid and thyroid hormone receptors (SMRT) repressor complex [6]. *TBL1XR1* is widely expressed throughout the body [6]. Although its full range of functions and interactions are not known, Tbl1xr1 has been implicated in a variety of cancer types including lymphoma [7], as well as in a spectrum of neurodevelopmental disorders [8, 9]. Although cancer and *TBL1XR1* neurodevelopmental disorder have not been reported to co-occur, overlapping genetic variants have been reported across both phenotypes [7].

*TBL1XR1* was first implicated as the genetic basis for Pierpont Syndrome, a rare disorder characterized by global developmental delay, epilepsy, feeding difficulties, and characteristic dysmorphic features including abnormal subcutaneous fat distribution with prominent plantar and digital fat pads and deep palmar and plantar grooves with “pillowing” of the palms and soles, along with microcephaly, midface hypoplasia, other facial anomalies, and short stature [10–14]. Pierpont Syndrome is linked to a specific heterozygous variant in *TBL1XR1* (c.1337A>G, p.Tyr446Cys) [10], although a limited number of additional variants associated with the phenotype have since been described [15, 16]. These variants have in common their location on the inner ring of the protein structure formed by the WD40 repeats [10, 15], leading to a proposed dominant negative effect altering protein–protein interactions with yet unknown downstream effects [10].

The phenotypic spectrum of *TBL1XR1*-related disorder, however, extends beyond Pierpont Syndrome. Recent reports have implicated *TBL1XR1* in a range of disorders including ASD, ID, epilepsy (including West Syndrome), attention-deficit/hyperactivity disorder (ADHD), and schizophrenia, with reported genetic variants ranging from typically heterozygous, de novo missense variants to microdeletions and microduplications [8, 9, 17–28]. Despite some similarities in presentation, this broader group of patients lacks the characteristic dysmorphism associated with Pierpont Syndrome and includes diagnoses such as ASD that were not previously reported in patients with Pierpont Syndrome. Although significant genetic and phenotypic heterogeneity has been reported in *TBL1XR1*-related neurologic disease, the effect of genotype on ultimate presentation is not clear. A relationship to Rett syndrome has been suggested due to the observation of some *TBL1XR1* missense mutations preventing MeCP2 binding [29]. However, defects in *TBL1XR1* are not associated with a uniform clinical picture but seem to vary between specific mutations [30]. Indeed, certain variants in the gene have been reported to produce the characteristic dysmorphism associated with Pierpont Syndrome while others produce neurodevelopmental sequelae with different or entirely absent dysmorphic features. As variants outside of the p.Tyr446 site have typically been reported only in single cases, it is unclear whether significant phenotypic variability can be associated with a single genetic variant.

These differences may point to distinct mechanisms of action between these presentations. It has previously been shown that the canonical Pierpont Syndrome variant (c.1337A>G, p.Tyr446Cys) assembles appropriately into the NCoR/SMRT complex, implying that the pathogenicity comes not from altered formation of the complex but more likely from altered protein–protein interactions and ultimately misdirection of the complex [10]. It is unknown whether other missense variants affect the protein in the same or a similar manner, which interactions are being altered, and how this may differ from pure loss of function variants such as deletions. It remains unclear to what extent patient genotype influences the resultant phenotype.

Understanding of the neurologic disease associated with *TBL1XR1* is currently limited due to the rare nature of this disorder and the heterogeneity in the range of phenotypes reported. The literature is primarily limited to case reports and small case series, with a lack of larger standardized cohorts and longitudinal data. Data from the perspective of families/caregivers, including their experiences and perceptions related to the disorder, have also been lacking.

Given the reported phenotypic variability, there is value in examining a larger population to determine the longitudinal course and developmental trajectory in these patients to enable a better understanding of prognosis and to create a natural history of the disorder that will inform clinical intervention trials. To address this need, we have conducted a survey of families/caregivers of patients with *TBL1XR1-*related disorder recruited via two online communities. The aim of this current study is to evaluate the phenotypic spectrum and developmental trajectories of patients with *TBL1XR1*-associated neurologic disease. Further, this study intends to examine the relationship between the array of underlying genetic variants and phenotypes seen in individual patients.

## Methods

### Participants

Participants were recruited via two pre-existing, parent-run closed Facebook groups devoted to families of patients with *TBL1XR1-*related disorder. A link to the survey was distributed to all members of both the *TBL1XR1* Gene Mutation Forum and the Pierpont Syndrome Facebook groups. While there are no formal criteria for membership in either group, the Pierpont Syndrome group is targeted towards individuals who have received a specific diagnosis of Pierpont Syndrome, while the *TBL1XR1* Gene Mutation Forum is more broadly inclusive of all individuals with *TBL1XR1*-related disorder. Participants of all ages and genotypes were eligible. Participants were eligible if they were English-speaking caregivers (parent or guardian) of a child with a *TBL1XR1* genetic mutation, as the survey materials were only available in English.

### Data collection

A survey was developed at Massachusetts General Hospital (MGH) using previously-published information on Pierpont Syndrome and *TBL1XR1*-related disorder, as well as clinical expertise in related disorders [10, 11, 13–15]. The survey was implemented on REDCap (Research Electronic Data Capture) and distributed with assistance from a parent active on both the *TBL1XR1* Gene Mutation Forum and the Pierpont Syndrome Facebook groups. A letter was posted inviting parents to participate in the survey and included a link to the REDCap survey. The study was approved by the Mass General Brigham Human Research Committee (Protocol #2020P003345).

The survey consisted of 14 subsections (Demographics, Family History, Pregnancy, Diagnosis of *TBL1XR1* Gene Mutation, Gross Motor Development, Fine Motor Development, Feeding, Language and Behavior, Eye Movements, Other Neurologic Findings, Autistic Behaviors, ADHD Behaviors, Diagnostic Work-Up, and Interventions). Once started, surveys were assigned a unique study number used for identification. Participants were provided with a password to use to return to the saved survey if not completed at one time. The survey was estimated to take 2–2.5 h to complete. Participants were able to submit genetic testing reports to confirm their reported variants. A follow-up survey was created to allow respondents to add genetic information that was not reported in the initial survey, although no new genetic information was collected in this way. Study data were collected and managed using REDCap electronic data capture tools hosted at MGH [31, 32].

### Statistics and visualization

Descriptive statistics were used to summarize the clinical features of subjects from the study population. Cumulative incidence was used to chart the probability of acquiring developmental milestones, experiencing regression, or experiencing seizures by age. The reported genetic variants were mapped onto the protein structure using the “lollipops” tool [33].

Preliminary results were presented at both the American College of Medical Genetics and Child Neurology Society 2022 Annual Meetings [34, 35].

## Results

### Participants

Between January 2021 and January 2023, 132 caregiver surveys were started. Ninety-one incomplete responses were excluded from analysis. This included all responses that were not marked as complete by the respondent, the majority of which were left entirely or mostly blank. It is suspected that these were accidental or duplicate entries from respondents who returned at a later time and started a new survey rather than using the previously-provided password to access and edit their original survey. Forty-one unique surveys, each describing one subject with *TBL1XR1*-related disorder, were fully submitted, and these were included for analysis. Of these, nine respondents (21.9%) did not provide genetic information and were included due to a self-reported diagnosis of *TBL1XR1*-related disorder or Pierpont Syndrome. The median age of the 41 included subjects was 8 years (range 1–25 years), and 53.7% were male. Genetic diagnoses had been made at a median age of 5 years, although the age at diagnosis ranged from 3 months to 16.5 years. Two subjects were twins and one was adopted. The demographic data for subjects is shown in Table 1.

**Table 1:** Sample characteristics (N = 41)

	n	Percentage (%)
Female	19	46.3
Male	22	53.7
Age		
0–5 years	12	29.3
6–10 years	18	43.9
11–15 years	2	4.9
16–20 years	5	12.2
21–25 years	3	7.3
Not reported	1	2.4

Median age at diagnosis 5 years (range 3 months–16.5 years)

### Pregnancy and perinatal period

Sixteen respondents (39.0%) endorsed complications during the pregnancy, including eight (19.5%) who reported issues with the fetus including poor growth, bilateral club foot, renal anomalies, decreased fetal movement, bradycardia, and abnormal nuchal translucency. Nine respondents (22.0%) delivered pre-term. Twenty-four (58.5%) reported complications in the perinatal period, most commonly admission to the neonatal intensive care unit (n = 15, 36.6%), followed by a need for supplemental oxygen (n = 9, 22.0%) and feeding difficulty/weight loss (n = 8, 19.5%). The infants were hospitalized for a median of 3.5 days following birth.

### Initial symptoms prompting genetic testing and neurological diagnoses

The most commonly endorsed symptoms prompting genetic testing were “not making developmental milestones” (n = 36, 87.8%), “low muscle tone (hypotonia)” (n = 32, 78.0%), “feeding difficulties” (n = 28, 68.3%), “impaired fine motor skills (such as holding a bottle)” (n = 26, 63.4%), “failure to thrive” (n = 23, 56.1%), and “sleep disturbance” (n = 20, 48.8%). Interestingly, thirty individuals (73.2%) received a distinct neurologic diagnosis prior to their genetic diagnosis. These initial neurologic diagnoses included ASD (n = 13, 31.7%), Chiari malformations (n = 8, 19.5%), epilepsy (n = 6, 14.6%), developmental issues (n = 5, 12.2%), ADHD (n = 3, 7.3%), hearing loss (n = 1, 2.4%), and hydrocephalus (n = 1, 2.4%). The most commonly endorsed symptoms leading to these initial neurologic diagnoses were “not making developmental milestones” (n = 25, 61.0%), “low muscle tone (hypotonia)” (n = 22, 53.7%), “failure to thrive” (n = 20, 48.8%), “issues with attention” (n = 19, 46.3%), “sleep disturbance (n = 18, 43.9%), “impaired fine motor skills (such as holding a bottle)” (n = 18, 43.9%), “feeding difficulties” (n = 18, 43.9%), and “autism symptoms” (n = 17, 41.5%).

### Genetics

**Fig. 1:**
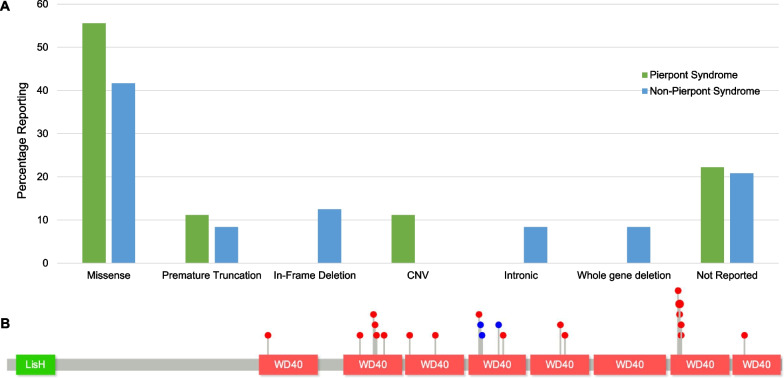
Genetic variants reported by survey participants (N = 41). **A** Types of genetic variants reported in patients diagnosed with Pierpont Syndrome (n = 18) compared to those without a Pierpont diagnosis (n = 23). Genetic information was not provided for 9 subjects. **B** Location of missense variants (red) and in-frame deletions (blue) in the *TBL1XR1* gene reported by survey participants. Recurrent sites are denoted by increased size of the “lollipop” circle. All missense variants (n = 20) and in-frame deletions (n = 3) reported by survey participants occurred in WD40 repeat regions

Thirty-three subjects (80.5%) underwent exome sequencing as part of their genetic evaluation. Genetic information was provided for 32 subjects (78.0%), one of whom had two separate genetic variants in *TBL1XR1* (Table 2). As the two variants in this individual affected adjacent codons, it is likely that these occurred as part of a single event but were automatically read as separate variants on the sequencing report. Of those that reported their genetic information, 26 had novel variants not reported in the prior literature, including premature truncation from frameshift and nonsense variants, in-frame deletions, missense variants, intronic variants, copy number variants (CNV), and whole gene deletions. Of the missense variants and in-frame deletions, all were found within the WD40 regions that are likely responsible for protein–protein binding (Fig. 1b).

**Table 2:** Genetic variants

Coding	Variant	Variant type	Responses	Pierpont Dx
*Premature termination*				
c.297dupT	p.R100*	Duplication/FS	1	Yes
c.327_357dup31	p.Q120Sfs*35	Duplication/FS	1	No
c.1588_1594dupGGCTGCA	p.T532fs	Duplication/FS	1	No
c.1524T>A	p.C508*	Nonsense	1	Yes
*In-frame deletion*				
c.943_945delGAT^1^	p.D315del	Deletion	1	No
c.941_943delTTG^1^	p.V314del	Deletion	1	No
c.977_979delGTA	p.S326del	Deletion	1	No
*Missense*				
c.519T>A	p.F173L	Missense	1	Yes
c.700A>G	p.N234D	Missense	1	Not sure
c.728G>A	p.G243D	Missense	1	No
c.730T>C	p.S244P	Missense	1	Yes
c.734A>G	p.Y245C	Missense	1	No
c.749G>A	p.R250K	Missense	1	No
c.800G>T	p.G267V	Missense	1	Not sure
c.851C>G	p.A284G	Missense	1	No
c.938A>C	p.D313A	Missense	1	No
c.987G>A	p.M329I	Missense	1	Yes
c.1100G>A	p.C367Y	Missense	1	Yes
c.1108G>A	p.D370N	Missense	1	Not sure
c.1333G>C	p.V445L	Missense	1	No
c.1336T>G	p.Y446D	Missense	1	Yes
c.1337A>G	p.Y446C	Missense	3	Yes
c.1340G>A	p.S447N	Missense	1	Yes
c.1341T>G	p.S447R	Missense	1	Not reported^2^
c.1466T>A	p.V489D	Missense	1	Yes
*Intronic*				
c.560+5G>C	IVS6+5G>C	Intronic	1	No
c.865-7A>G	IVS9-7A>G	Intronic	1	No
*CNV*				
3q26.32 (176.654.197–176.884.880) × 3		Duplication	2	Yes
3q26.31q26.32	Deletion (1.58 Mb)	Deletion	1	No
3q26.32q26.36	Deletion (25.71 kb)	Deletion	1	No

^1^Single subject carries both deletions

^2^No response entered

Eighteen subjects (43.9%) were diagnosed with Pierpont Syndrome, the majority of whom reported having at least some dysmorphic features characteristic of the disorder. In contrast to the prior literature linking Pierpont Syndrome to a small number of specific missense variants in *TBL1XR1* [8, 10, 15, 36], individuals in this survey received the diagnosis associated with a wide array of genetic variants in *TBL1XR1*, including other missense variants, premature truncation, and CNV (Fig. 1b). Genetic information was not reported for nine subjects, including four diagnosed with Pierpont Syndrome. Only three survey subjects were reported to carry a variant previously reported to cause Pierpont Syndrome (all with c.1337A>G, p.Tyr446Cys). In-frame deletions, intronic variants, and whole gene deletions were only seen in subjects not diagnosed with Pierpont Syndrome (Fig. 1b).

Fifteen subjects (36.6%) were reported to have a gene mutation beyond *TBL1XR1*. Of these, seven respondents provided their additional genetic findings, including two subjects who each had two additional genetic conditions reported (Additional file 1: Table S1). The majority of these did not provide the specific variant found nor did they provide variant pathogenicity or zygosity information, so it was not clear whether these were carriers or affected individuals. Notably, five respondents who reported an additional genetic finding listed only the subject’s *TBL1XR1* variant as the additional genetic finding.

### Developmental trajectory

**Fig. 2:**
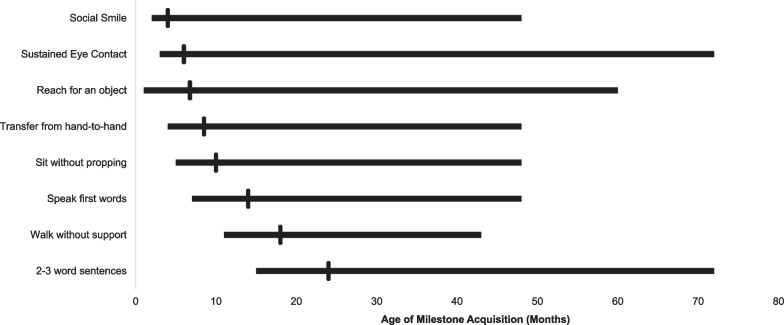
Developmental trajectory in patients with *TBL1XR1*-related disorder.. The age of reported developmental milestone acquisition is shown as a range with a vertical bar denoting the median

**Fig. 3:**
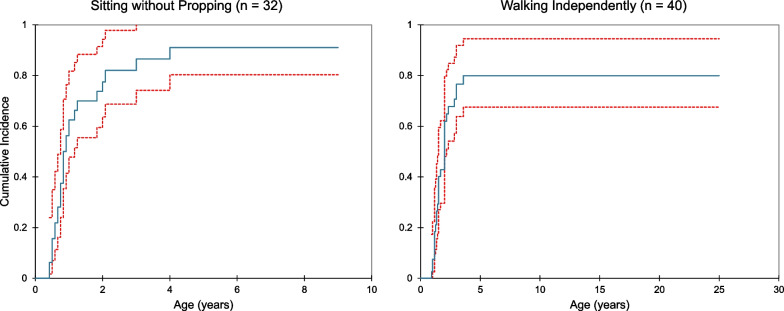
Acquisition of gross motor skills in patients with *TBL1XR1*-related disorder. Cumulative incidence curves are shown representing the age of acquisition of major motor milestones, with red lines denoting 95% confidence intervals. Respondents were excluded if the skill was attained but the age of acquisition was not reported or if it was unable to be determined whether the skill was obtained

**Fig. 4:**
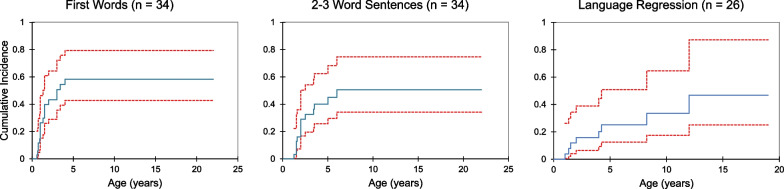
Gain and loss of language skills in patients with *TBL1XR1*-related disorder. Cumulative incidence curves are shown representing the age of acquisition and loss of major language milestones, with red lines denoting 95% confidence intervals. Respondents were excluded if the skill was attained or regression occurred but the age of acquisition or regression was not reported. Respondents were also excluded if it was unable to be determined whether the skill was obtained or whether regression occurred

Subjects experienced a range of developmental outcomes from near-normal to severe delay apparent in the first years of life (Fig. 2). Language acquisition appeared most significantly affected, with only 61% (n = 25) acquiring first words and 46% (n = 19) speaking in 2–3 word sentences (Table 3). In contrast, 71% of the population (n = 29) was able to walk independently at some point, with an even larger proportion acquiring earlier motor milestones. Cumulative incidence curves are shown in Figs. 3 and 4, adjusting the probability of milestone acquisition and regression for the young age of the cohort. Notably, those who reported milestone acquisition or regression but did not provide an age of acquisition or regression were excluded from these analyses. Also excluded were those for whom there was not sufficient information provided to determine whether milestones were acquired or regression occurred. Although developmental regression occurred in a minority of subjects, it was most common in the language domain, affecting 10 subjects by the second decade of life. Five individuals experienced motor regression, and social regression was seen in 3 individuals. Of the 14 individuals reporting regression in any domain, seven (50%) had also reported perinatal complications, which is slightly less than the percentage with perinatal complications in the overall cohort (n = 24, 58.5%). The onset of regression was reported at a wide range of ages, from the first year of life until late in the second decade*.* The majority of caregivers (90–100% per question) provided responses regarding milestone acquisition and regression. Fewer, however, provided the subject’s age at the time of acquisition and regression. A minority of respondents provided the age of acquisition for social milestones (social smile and sustained eye contact).

**Table 3:** Milestone acquisition and regression

Developmental milestone	Number acquired n (%)	Number regressed n (%)	Acquisition age (mo) Median (range)	Regression age (mo) Median (range)
Social smile	38 (93)	3 (8)	4 (2–48)	12 (7–204)
Sustained eye contact	30 (73)	1 (3)	6 (3–72)	12 (12)
Reach for an object	39 (95)	0 (0)	6 (1–60)	N/A
Transfer from hand to hand	35 (85)	1 (3)	9 (4–48)	NR
Sit without propping	34 (83)	3 (9)	10 (5–48)	48 (7–192)
Speak first words	25 (61)	10 (40)^a^	14 (7–48)	36 (12–144)^a^
2–3 word sentences	19 (46)		24 (15–72)	
Walk without support	29 (71)	1 (3)	18 (11–43)	NR
Grasp (regression only)	NR	4 (10)^b^	NR	24 (12–36)

^a^Language regression was not broken down by skill and included as a general loss of language

^b^Percentage reported out of total sample as number with acquisition unknown

N/A—not applicable; NR—not reported

### Seizures

**Fig. 5:**
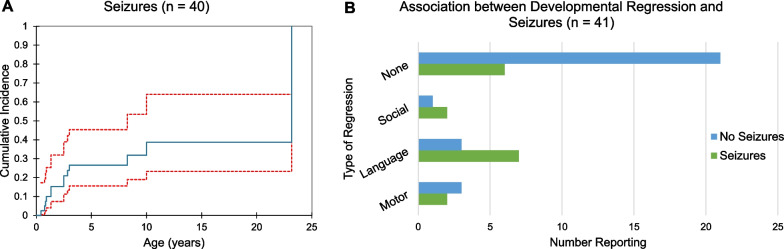
The presence of seizures and association with developmental regression in patients with *TBL1XR1*-related disorder. **A** Cumulative incidence curve is shown representing the onset of seizures in 13 patients by age, with red lines denoting the 95% confidence interval. Respondents were excluded if age at the onset of seizures was not reported. **B** The presence of regression is shown by developmental domain, with those reporting no regression indicated by ‘none’. Groups are further divided by the presence or absence of seizures

Fourteen subjects (34.1%) experienced seizures (median age at onset 2.5 years, range 0.4–23.2 years). Individuals with seizures were more likely to have experienced perinatal complications (64.2%, n = 9, v. 55.6%, n = 15, of those without seizures). Reported seizure types included absence, tonic–clonic, focal, tonic, and epileptic spasms. The majority of those who developed seizures did so before the age of 10 years (Fig. 5a). Notably, although the majority of respondents indicated some degree of seizure control (ranging from partial to complete) on medication, all but one subject for whom data was available reported experiencing seizure(s) in the past 2 years (Additional file 2: Table S2). For five subjects, however, timing of the most recent seizure could not be determined.

As epilepsy is a known cause of developmental regression, the relationship between seizures and regression in this population was examined. Half of those with seizures (n = 7, 50.0%) also endorsed language regression (Fig. 5b). There was no significant difference between the severity of language delay in the cohort with and without seizures. The relationship between seizures and motor or social regression was less apparent, with two patients with epilepsy reporting gross motor regression (of a total of 5 individuals with motor regression) and two reporting social regression (of 3 total individuals with social regression). There was no relationship between the reported seizure type and the presence of developmental regression.

### Associated signs and symptoms

The majority of subjects experienced developmental delay, feeding problems, hypotonia, failure to thrive, and sleep disturbance at some point in their course, even if not currently ongoing. Chiari malformations and spine issues, including scoliosis, were both frequently reported (n = 9, 22.0%, and n = 8, 19.5%, respectively). Clinically diagnosed movement disorders, such as ataxia (n = 4, 9.8%) or dyskinesia (n = 1, 2.4%), were less commonly reported.

ASD and ADHD were common diagnoses affecting the survey population, with self-reported diagnoses in 39.0% (n = 16) and 29.3% (n = 12) respectively. Certain behaviors characteristic of these disorders were widespread, affecting even a proportion of respondents without a formal diagnosis. These behaviors included impulsivity (n = 20, 48.8%), repetitive behaviors (n = 20, 48.8%), climbing and standing on objects whenever possible (n = 18, 43.9%), becoming upset by everyday noises, clothing, and smells (n = 15, 36.6%), and self-injurious behaviors (n = 15, 36.6%).

### Feeding

Most respondents (n = 36, 87.8%) endorsed feeding difficulties, with a median age of onset at 1 month of age (range 0–192 months). Thickened feeds were commonly needed (n = 16, 39.0%), and a subset of subjects required partial or complete tube feeds at some point in their course. Seventeen individuals reported having a nasogastric tube placed, with the majority also reporting perinatal complications (n = 12, 70.6%), Similarly, six of the ten individuals requiring ongoing partial or complete tube feeding had perinatal complications. Associated gastrointestinal diagnoses were common, most frequently gastroesophageal reflux disease (n = 18, 43.9%), but also including constipation (n = 4, 9.8%), dysmotility/gastroparesis (n = 3, 7.3%), swallowing difficulties (n = 3, 7.3%), structural issues (n = 2, 4.9%), H. Pylori (n = 1, 2.4%), and chronic emesis (n = 1, 2.4%). Growth issues were common (n = 26, 63.4%), with weight loss affecting 24.4% (n = 10) of subjects.

## Discussion

We report here the results of a caregiver survey describing the largest cohort of patients with *TBL1XR1*-related disorder to date. In this cross-sectional study, we analyzed the pregnancy and perinatal course, caregiver-reported developmental trajectory, associated symptoms and diagnoses, and neurological progression over time as well as genetic information of 41 subjects with *TBL1XR1*-related disorder.

Nearly three-quarters of patients with *TBL1XR1*-related disorder were diagnosed with a neurologic condition before they received their genetic diagnosis. The high prevalence of neurologic diagnoses in this population highlights the importance of raising provider awareness of the condition and the need to undergo appropriate genetic testing. This is particularly relevant in the case of ASD, as nearly one-third of the study population were diagnosed initially with ASD. Many of the early presenting signs and symptoms reported by caregivers were nonspecific, such as developmental delay, hypotonia, and growth issues, highlighting the importance of inclusion of *TBL1XR1* on gene panels. Of note, a significant number of patients experienced perinatal complications. While the underlying genetic diagnosis likely predisposed patients to these complications, we cannot rule out that differences in medical management and clinical events contributed independently to brain injury and the longitudinal disease course.

Genetic information was provided by over three-quarters of respondents. Most of the genetic variants reported in this study had not been described in prior literature. Further, the subjects diagnosed with Pierpont Syndrome carried variants in *TBL1XR1* beyond those that had been previously reported in association with Pierpont Syndrome. The variants reported in association with Pierpont Syndrome included frameshift, nonsense, missense, and whole gene duplications, which was unexpected as these variants likely have different mechanisms of disease. As our study was limited to survey data without in-person assessments or photographs to assess dysmorphism directly, we were limited in our ability to corroborate a diagnosis of Pierpont Syndrome. While the majority of those who were diagnosed with Pierpont Syndrome were reported to have at least some of the classic features, it is possible that some of those reporting a Pierpont diagnosis may not truly fit with the clinical phenotype and were instead diagnosed solely due to their genetic findings. Further study is needed, but this raises the possibility that features of Pierpont Syndrome can be seen more broadly with other changes in the *TBL1XR1* gene.

It is noteworthy, however, that all variants suspected to exert a local effect on the gene (i.e. missense mutations and in-frame deletions) were found in the WD40 repeat regions (Fig. 1b). These regions have previously been shown to be responsible for protein–protein interactions with an as-yet unknown interaction partner, which may mediate the neurologic phenotype. Despite their common localization to the WD40 repeat regions, these variants ultimately led to a range of phenotypic features, encompassing both Pierpont Syndrome and non-Pierpont presentations. It is not clear whether this is due to differing effects of these variants on protein–protein interactions related to the 3D structure of Tbl1xr1 or whether other compensatory mechanisms might mediate this phenotypic difference. Notably, *TBL1XR1* is predicted to be poorly tolerant of loss-of function variants, with a loss-of-function observed/expected upper bound fraction (LOEUF) score of 0.11 and none reported in the Genome Aggregation Database (gnomAD) [37]. This supports the pathogenicity of such variants and the clustering of our variants suggests the importance of the WD40 regions, but it does not explain the phenotypic differences.

Further, it is notable that when acquired as somatic rather than germline changes, the same genetic variants that lead to neurodevelopmental impairment are also implicated in the cancer phenotype [7]. Indeed, the overall variant profile is similar between those reported in diffuse large B-cell lymphomas (DLBCL) and the neurologic phenotype. In DLBCL, the D370Y variant has been shown to alter interactions with the SMRT/HDAC3 complex [7]. It is possible that a similar alteration occurs in the neurologic phenotype. One limitation in the data is the relatively large proportion of respondents who did not provide genetic information, as this limits the ability to make genotype–phenotype correlations. It is not known whether functional differences caused by different variants in *TBL1XR1* may explain the phenotypic differences seen here. Our data can now pave the way for more systematic experimental evaluation of altered protein–protein interactions across a range of mutations in *TBL1XR1*. An example of this process is known as “edgotyping” [38].

It is notable that a large proportion of respondents (n = 15, 36.6%) reported receiving additional genetic findings in addition to those related to *TBL1XR1*. As the information provided by most respondents was limited, it was not possible to determine whether these were diagnosed genetic conditions or if some were benign variants or carriers only. Further, five respondents listed the *TBL1XR1* variant in this field, so it is not clear whether these subjects truly had additional genetic findings. Regardless, one participant reported the presence of both 47, XYY and compound heterozygous variants in *FLG*, making it likely that the clinical variability associated with *TBL1XR1*-related disorder may be confounded to some degree by these secondary genetic findings.

The vast majority of respondents provided information regarding age of developmental milestone acquisition and loss, as applicable. The responses revealed a range of developmental outcomes, from near-normal to severe delay and, in a minority of cases, developmental regression. Although delays and regression were seen across developmental domains, language appeared most significantly affected with the smallest percentage of the cohort achieving more advanced language milestones. Loss of language skills was also the most frequently reported type of regression.

Seizures were seen in over one-third of subjects, frequently starting within the first years of life and nearly always before the age of ten. The majority of respondents had a favorable response to medication; however, most had experienced a seizure within the 2 years prior to survey completion. This suggests that although there may be a degree of seizure control, it is too early to determine whether this will be long-lasting. Language regression was seen in half of the subjects with seizures despite occurring in slightly less than a quarter of the total respondents. Language may be directly impacted by the occurrence of seizures or the anticonvulsants used but language regression and seizures may also be part of a larger neurodegenerative process without a direct causal link. The potential relationship between seizures and language regression in this population was not seen in other developmental domains, with no apparent relationship between seizures and motor or social regression. The interplay between seizures and developmental regression is not fully explained and remains an area for future study. Perinatal complications were reported at similar rates in those that experienced regression as in the overall cohort, making this less likely to explain the developmental differences.

Both ASD and ADHD were commonly diagnosed in this population. Behaviors associated with these diagnoses, such as impulsivity and repetitive behaviors, were reported more frequently than were formal diagnoses, suggesting that relying on diagnosis alone may underrepresent the prevalence of behavioral issues. Given the prevalence of ASD and ADHD in this population, all patients with *TBL1XR1*-related disorder warrant careful screening for these conditions. Whether the presence of these diagnoses is associated with a more severe phenotype cannot be determined given the small sample size and is an area of future study. Other actionable conditions, such as sleep disturbances, Chiari malformations, and spine issues were reported and would benefit from clinical consideration.

Beyond the associated neurologic impairment, the majority of subjects endorsed feeding difficulties. These difficulties ranged from aspiration requiring thickened feeds and, in a subset of patients, partial or complete tube feeds, to reflux and dysmotility. A majority of patients reported growth issues. The high prevalence of gastrointestinal and growth issues in this population should prompt close clinical monitoring and referral for specialist feeding and nutritional support when needed.

Limitations of the study lie in its cross-sectional, caregiver-reported nature and the small sample size under study. Although the vast majority of caregivers reported on developmental milestone acquisition and regression, far fewer reported associated ages. These data are limited by caregiver recollection and inability to be objectively verified. There may be variance in caregiver ability to recall less discrete skills, such as making sustained eye contact, which can lead to bias in the reporting. Inconsistent responses in the survey data can lead to limitations in interpretation. The data are further limited by the relatively young age of the subjects, making it difficult to draw conclusions regarding events that may arise later in life, such as seizures, that may not have yet occurred at the time of survey completion. This may lead to underestimating the frequency of some events in the populations. We have attempted to account for this by using cumulative incidence to adjust for the younger age of participants. Finally, participants were recruited from two disease-specific Facebook groups. This may lead to selection bias, as those who participate in the Facebook groups and engage with the survey are likely more actively involved in the community and may differ from other affected families in ways that affect the reported characteristics.

It is only recently that *TBL1XR1* has been linked to neurologic disease. This recognition has led to its inclusion on disease-specific gene panels by major genetic testing companies, although the timing of inclusion and indications for testing are inconsistent between companies. *TBL1XR1* was initially added to Invitae epilepsy panels in 2016, while GeneDx added it to the test menu in 2018. As awareness of the *TBL1XR1* phenotype has expanded, it has been added to a wider array of panels including those for neurodevelopmental disorders, ASD, ID, cerebral palsy, and hypotonia. As genes are added to particular panels, this can bias diagnosis towards those with particular clinical features and away from other presentations, over time affecting the apparent natural history of the disease. Further, not all aspects of the disease course may be related to the genetic defect in *TBL1XR1*. We found a significant number of patients experiencing perinatal complications and these could certainly be contributing to the reported course of the disease. Additional genetic diagnoses were additionally reported in a significant proportion of the population, and it is not clear the extent to which this contributes to the reported phenotypes.

Given the timing of diagnoses in this cohort, most of the subjects likely would not have been diagnosed based on genetic panels. Indeed, 33 subjects (80.5%) reported undergoing exome sequencing. While exome and genome sequencing allow for a broader examination of genetic variants with less bias towards one specific clinical presentation, they require careful clinical phenotyping and may require reanalysis over time as new candidate genes are identified and phenotypic features are better elucidated.

To better understand *TBL1XR1*-related disorder, it is helpful to view it in the context of other genetic causes of ASD and ID. While these disorders share many common features, each carries distinct phenotypic features that allow for clinical recognition (Table 4). While developmental delay is nearly universal in *TBL1XR1*-related disorder, regression occurs in one-third only and most commonly affects language function. This is in contrast to related disorders such as Angelman Syndrome, where regression is not typically seen [39], or disorders with more common motor or social regression, such as Rett Syndrome [40, 41]. The triggers and mechanisms underlying regression due to *TBL1XR1* gene defects are currently unknown. While the diverse nature of WD40 interactions could play a role [10], a relationship remains speculative at this early stage in the field. Nearly one-third of subjects with *TBL1XR1-*related disorder in the current survey reported seizures, a lower prevalence than is seen in Angelman Syndrome [39] and Rett Syndrome [40], but similar to related disorders such as Phelan-McDermid syndrome [42]. Although only seen in a subset of patients (classically those diagnosed with Pierpont Syndrome), the abnormal subcutaneous fat distribution and dysmorphic facies characteristic of this disorder remain a distinguishing feature.

**Table 4:** Comparison of genetic syndromes associated with autism spectrum disorder

Disorder	Gene	Inheritance	ASD	Regression	Seizures	GI issues	Sleep	Behavioral issues		Systemic involvement	Unique features
								Self-injury	Repetitive behaviors		
*TBL1XR1*/Pierpont syndrome^a^	*TBL1XR1*	AD	++	++	++	+++	+++	++	++	Renal and cardiac anomalies, anal malformation	Fetal fat pads, high forehead
Phelan McDermid syndrome	*SHANK3*	AD	++++	+++	++	++	++	++	++++	Renal and cardiac anomalies, hypothyroidism	Heat intolerance, fleshy hands, dysplastic toenails
Pitt-Hopkins syndrome	*TCF4*	AD	+++	–	++	++++	+	+++	++++	Paradoxical breathing pattern	Coarse facial features, overriding toes, fetal fat pads, peaked philtrum
Fragile X syndrome	*FMR1*	XLD	++	–	+	++	++	+++	+++	Macroorchidism cardiac anomalies	Long face, large/prominent ears, high-arched palate, and prominent jaw
Rett syndrome	*MECP2*	XLD	++/+++	++++	+++/+++	++	++	++/+++	++++	Paradoxical breathing pattern, vision, cardiac involvement	Stereotypic hand movement, loss of purposeful hand movement
Angelman syndrome	*UBE3A*	AD	+++	–	++++	++++	++++	++	+/++++	Vision	Happy disposition, frequent laughter, excitable personality

^a^Data taken from this survey

Frequency of features reported in surveys (*TBL1XR1*) or available literature (related disorders) is denoted as follows: + < 25%; ++ 25–49%; +++ 50 = 74%; ++++ >  = 75%; –absent

Frequency of features associated with related genetic syndromes associated with autism spectrum disorder obtained from review of prior literature. Phelan McDermid Syndrome: Frank Y. The Neurological Manifestations of Phelan-McDermid Syndrome. Pediatr Neurol. 2021 Sep;122:59–64. Soorya L, Kolevzon A, Zweifach J, Lim T, Dobry Y, Schwartz L, et al. Prospective investigation of autism and genotype–phenotype correlations in 22q13 deletion syndrome and SHANK3 deficiency. Mol Autism. 2013 Jun 11;4:18. Pitt-Hopkins Syndrome: Goodspeed K, Newsom C, Morris MA, Powell C, Evans P, Golla S. Pitt-Hopkins Syndrome: A Review of Current Literature, Clinical Approach, and 23-Patient Case Series. J Child Neurol. 2018 Mar;33(3):233–44. Watkins A, Bissell S, Moss J, Oliver C, Clayton-Smith J, Haye L, et al. Behavioural and psychological characteristics in Pitt-Hopkins syndrome: a comparison with Angelman and Cornelia de Lange syndromes. J Neurodev Disord. 2019 Oct 5;11(1):24. Fragile X Syndrome: Oakes A, Thurman AJ, McDuffie A, Bullard LM, Hagerman RJ, Abbeduto L. Characterising repetitive behaviours in young boys with fragile X syndrome. J Intellect Disabil Res. 2016;60(1):54–67. Ciaccio C, Fontana L, Milani D, Tabano S, Miozzo M, Esposito S. Fragile X syndrome: a review of clinical and molecular diagnoses. Ital J Pediatr. 2017 Dec;43(1):39. Arron K, Oliver C, Moss J, Berg K, Burbidge C. The prevalence and phenomenology of self-injurious and aggressive behaviour in genetic syndromes. J Intellect Disabil Res. 2011;55(2):109–20. Moss J, Oliver C, Arron K, Burbidge C, Berg K. The Prevalence and Phenomenology of Repetitive Behavior in Genetic Syndromes. J Autism Dev Disord. 2009 Apr;39(4):572–88. Rett Syndrome: Gold WA, Krishnarajy R, Ellaway C, Christodoulou J. Rett Syndrome: A Genetic Update and Clinical Review Focusing on Comorbidities. ACS Chem Neurosci. 2018 Feb 21;9(2):167–76. Frullanti E, Papa FT, Grillo E, Clarke A, Ben-Zeev B, Pineda M, et al. Analysis of the Phenotypes in the Rett Networked Database. Int J Genomics. 2019 Mar 27;2019:6,956,934. Wulffaert J, Van Berckelaer-Onnes IA, Scholte EM. Autistic disorder symptoms in Rett syndrome. Autism. 2009 Nov 1;13(6):567–81. Cianfaglione R, Clarke A, Kerr M, Hastings RP, Oliver C, Moss J, et al. A national survey of Rett syndrome: behavioural characteristics. J Neurodev Disord. 2015 Mar 4;7(1):11. Angelman Syndrome: Bird LM. Angelman syndrome: review of clinical and molecular aspects. Appl Clin Genet. 2014 May 16;7:93–104. Glassman LW, Grocott OR, Kunz PA, Larson AM, Zella G, Ganguli K, et al. Prevalence of gastrointestinal symptoms in Angelman syndrome. Am J Med Genet A. 2017;173(10):2703–9. Larson AM, Shinnick JE, Shaaya EA, Thiele EA, Thibert RL. Angelman syndrome in adulthood. Am J Med Genet A. 2015 Feb;167(2):331–44

## Conclusions

*TBL1XR1*-related disorder encompasses a spectrum of clinical presentations, marked by early developmental delay ranging in severity, with a subset of patients experiencing developmental regression towards the second decade. Seizures are common and may be related to language regression, although were not clearly related to regression in the motor and social domains. Further study is needed to determine whether functional differences caused by different variants in *TBL1XR1* explain the phenotypic differences.

### Supplementary Information

**Additional file 1: Table S1.** Genetic Findings beyond *TBL1XR1* reported by survey respondents. Description of data: This table lists the additional genetic findings beyond those affecting the *TBL1XR1* gene reported by survey respondents.

**Additional file 2: Table S2.** Medications taken in connection with *TBL1XR1*-related disorder. Description of data: The table lists the medications taken by patients in connection with *TBL1XR1*-related disorder, including medication class, the number of participants reporting, and side effects. For anticonvulsants, the effect on seizure frequency is listed.

## Data Availability

As *TBL1XR1-*related disorder is ultra rare and information included in the caregiver surveys may be personally identifying in this content, raw data from caregiver surveys are not publicly available to preserve individuals’ privacy. The datasets used during the current study are available from the corresponding author on reasonable request.

## Abbreviations

ADHD: Attention-deficit/hyperactivity disorder
ASD: Autism spectrum disorder
CNV: Copy number variant
DLBCL: Diffuse large B-cell lymphoma
ID: Intellectual disability
MGH: Massachusetts General Hospital
NCoR: Nuclear receptor corepressor
REDCap: Research electronic data capture
SMRT: Silencing mediator of retinoic acid and thyroid hormone receptors
TBL1XR1: Transducin (beta)-like X-linked receptor 1
