# Supplementary material for: The spectrum of neurological presentation in individuals affected by TBL1XR1 gene defects
Source: Orphanet J Rare Dis. 2024 Feb 20;19:79. doi: 10.1186/s13023-024-03083-3 (PMC10880200; doi:10.1186/s13023-024-03083-3)
Supplement: Supplementary file 1 — Additional file 1: Table S1. Genetic Findings beyond TBL1XR1 reported by survey respondents. Description of data: This table lists the additional genetic findings beyond those affecting the TBL1XR1 gene reported by survey respondents. [file 13023_2024_3083_MOESM2_ESM.docx]

**Additional File 2**

| **Table S2:** Medications taken in connection with *TBL1XR1*-related disorder | | | | |
| --- | --- | --- | --- | --- |
| Class | Medication | Number Taking | Effect on Seizures | Side Effects |
| Anticonvulsants | Benzodiazepines | 3 | + (1), unknown (2) | None reported |
|  | Levetiracetam | 5 | NC (1) unknown (3) | Severe drowsiness (1) |
|  | Cannabidiol | 3 | + (2) unknown (1) | None reported |
|  | Lacosamide | 2 | NC | None reported |
|  | Clobazam | 2 | + | Drowsiness (1) |
|  | Carbemazepine | 1 | + | None reported |
|  | Lamotrigine | 2 | NR | Mild drowsiness (1) |
|  | Oxcarbazepine | 1 | NR | Weight gain (1) |
|  | Perampanel | 1 | - | Erratic behavior (1), seizures worsened (1) |
|  | Topiramate | 1 | NR | Kidney stones (1) |
|  | Valproic Acid | 2 | + | Drowsiness (1) |
|  | Zonisamide | 1 | - | Seizures worsened (1) |
| Antipsychotics | Risperidone | 3 |  | Sedation (2), increased appetite (1) |
|  | Quetiapine | 1 |  | Sedation (1) |
| Antidepressants | SSRIs (fluoxetine, fluvoxamine, sertraline) | 3 |  | None reported |
| Antacids | Proton pump inhibitor (omeprazole, esomeprazole) | 6 |  | Anemia (1) |
|  | H2 antagonist (famotidine) | 2 |  | None reported |
| Laxatives | Polyethylene glycol | 7 |  | None reported |
|  | Lactulose | 1 |  | Vomiting (1) |
|  | Magnesium Hydroxide | 1 |  | None reported |
|  | Sodium Picosulfate | 1 |  | None reported |
|  | Enemas | 1 |  | None reported |
|  | Glycerin Suppository | 1 |  | None reported |
| Gut Motility Stimulator | Erythromycin | 2 |  | None reported |
|  | Azithromycin | 1 |  | None reported |
|  | Cyproheptadine | 3 |  | Sedation (1) |
|  | Metoclopramide | 1 |  | None reported |
|  | Domperidone | 1 |  | None reported |
| Sleeping aid | Melatonin | 5 |  | None reported |
| Tone Management | Carbidopa/Levodopa | 1 |  | None reported |
| Stimulant | Lisdexamfetamine | 1 |  | None reported |
|  | Methylphenidate | 1 |  | None reported |
| Alpha Agonist | Guanfacine | 2 |  | None reported |
|  | Clonidine | 2 |  | None reported |
| Endocrine | Somatropin | 1 |  | None reported |
|  | Levothyroxine | 1 |  | None reported |
|  | Hydrocortisone | 1 |  | None reported |
|  | Prednisolone | 1 |  | Severe diarrhea (1) |
| Antibiotics | Trimethoprim | 1 |  | None reported |
|  | Ciprofloxacin/dexamethasone | 1 |  | None reported |
| Diuretic | Furosemide | 1 |  | None reported |
| Asthma Management | Salbutamol (inhaler) | 1 |  | None reported |
|  | Montelukast | 1 |  | None reported |
|  | Budesonide / Formoterol (inhaler) | 1 |  | None reported |
| Supplements | Iron | 2 |  | None reported |
|  | Vitamin D | 2 |  | None reported |
|  | NAC | 1 |  | None reported |
|  | Multivitamin | 1 |  | None reported |
| Antihistamine | Loratadine | 1 |  | None reported |
|  | Diphenhydramine | 1 |  | None reported |
|  | Cetirizine | 1 |  | None reported |
| + Seizures decreased; - Seizures worsened; NC No change; NR Not reported | | | |  |
