# Supplementary material for: The spectrum of neurological presentation in individuals affected by TBL1XR1 gene defects
Source: Orphanet J Rare Dis. 2024 Feb 20;19:79. doi: 10.1186/s13023-024-03083-3 (PMC10880200; doi:10.1186/s13023-024-03083-3)
Supplement: Supplementary file 2 — Additional file 2: Table S2. Medications taken in connection with TBL1XR1-related disorder. Description of data: The table lists the medications taken by patients in connection with TBL1XR1-related disorder, including medication class, the number of participants reporting, and side effects. For anticonvulsants, the effect on seizure frequency is listed. [file 13023_2024_3083_MOESM1_ESM.docx]

| **Table S1:** Genetic Findings beyond *TBL1XR1* reported by survey respondents | | | |
| --- | --- | --- | --- |
| Gene/Condition^1^ | Coding Variant | Protein Change | Zygosity |
| *GJB2* | c.101T>C | p.M34T |  |
| *BRCA2*^2^ |  |  |  |
| *G6PD*^2^ |  |  |  |
| Lynch Syndrome |  |  |  |
| 15q11.2 |  |  |  |
| *TCF20* | c.2584G>C | p.Glu862Gln |  |
| *ATN1* | c.3521C>T | p.Ala1174Val | Heterozygous |
| 47, XYY^3^ |  |  |  |
| *FLG*^3^ |  |  | Compound heterozygous |

^1^Each genetic condition was reported by one respondent; blank fields were not reported

^2^*BRCA2* and *G6PD* were reported by the same participant.

^3^47, XYY and biallelic pathogenic variants in *FLG* were reported by the same participant.
